# Continuous monitoring in COVID-19 care: a retrospective study in time of crisis

**DOI:** 10.1093/jamiaopen/ooab030

**Published:** 2021-04-10

**Authors:** Roy de Ree, Jorn Willemsen, Gilbert te Grotenhuis, Rick de Ree, Joé Kolkert, Malou Peppelman

**Affiliations:** 1 Department of Health Innovation, Slingeland Hospital, Doetinchem, Gelderland, The Netherlands; 2 Department of Surgery, Slingeland Hospital, Doetinchem, Gelderland, The Netherlands

**Keywords:** COVID, telemedicine, remote sensing technology

## Abstract

**Background:**

A new monitoring system was implemented to support nursing staff and physicians on the COVID-19 ward. This system was designed to remotely monitor vital signs, to calculate an automated Early Warning Score, and to help identify patients at risk of deterioration.

**Methods:**

Hospitalized patients who tested positive for SARS-CoV-2 were connected to 2 wireless sensors measuring vital signs. Patients were divided into 2 groups based on the occurrence of adverse events during hospitalization. Heart and respiratory rate were monitored continuously and an automated EWS was calculated every 5 minutes. Data were compared between groups.

**Results:**

Prior to the occurrence of adverse events, significantly higher median heart and respiration rate and significantly lower median SPO_2_ values were observed. Mean and median automated EWS were significantly higher in patients with an adverse event.

**Conclusion:**

Continuous monitoring systems might help to detect clinical deterioration in COVID-19 patients at an earlier stage.

LAY SUMMARY

A new monitoring system was implemented to support nursing staff and physicians on the COVID-19 ward. This system was designed to remotely monitor vital signs, like respiratory rate, heart rate, and the oxygen level in the blood. These parameters were used to calculate an automated early warning score which helps to identify patients at risk of deterioration. Hospitalized patients who tested positive for SARS-CoV-2 were connected to two wireless sensors. Heart and respiratory rate were monitored continuously and an automated EWS was calculated every 5 minutes. Data were compared between patients at the COVID-19 ward and patients who were transported to the ICU or died. COVID patients at the ICU or those who died had significantly higher median heart and respiration rate and significantly lower median oxygen levels. These findings showed that continuous monitoring systems might help to detect clinical deterioration in COVID-19 patients at an earlier stage.

## BACKGROUND

Monitoring vital signs is essential in detecting clinical deterioration in hospitalized patients. The Early Warning Score (EWS) has proven to be a useful tool in identifying patients at risk of deterioration [1]. This bedside assessment of 6 physiological parameters (respiratory rate, oxygen saturation, temperature, blood pressure, pulse rate, and level of consciousness) provides the nursing staff a tool to evaluate the clinical condition of patients and notify rapid response teams (RRTs) quickly in case of imminent clinical instability. This might shorten the delay to intervention and improve patient outcome [2, 3]. However, manual monitoring is time consuming thus imposing a substantial burden on the nursing staff. Innovative sensor technology allows more frequent assessment of physiological parameters and, in theory, detects clinical deterioration in an earlier stage [4]. Several recent papers have shown that current wireless monitoring systems are capable of detecting changes in vital signs in patients who develop adverse events. These measurements correlate well with gold standard measurements [4–6]. However, due to the heterogeneity of systems and sensors used in different studies there is of yet lacking evidence supporting universal adoption of continuous monitoring [7, 8].

Different patient populations might need their own specific systems. We adjusted a monitoring system, originally designed to detect clinical deterioration in electively admitted patients to support nursing staff and physicians on the COVID-19 ward. This system was designed to remotely monitor vital signs (pulse rate, respiratory rate, and oxygen saturation), calculate an automatic Early Warning Score (aEWS) and used to try to identify COVID-19 patients at risk of deterioration. In this overview, we describe the system used, the lessons learned, and the challenges yet remaining.

## METHODS

**Figure 1.**
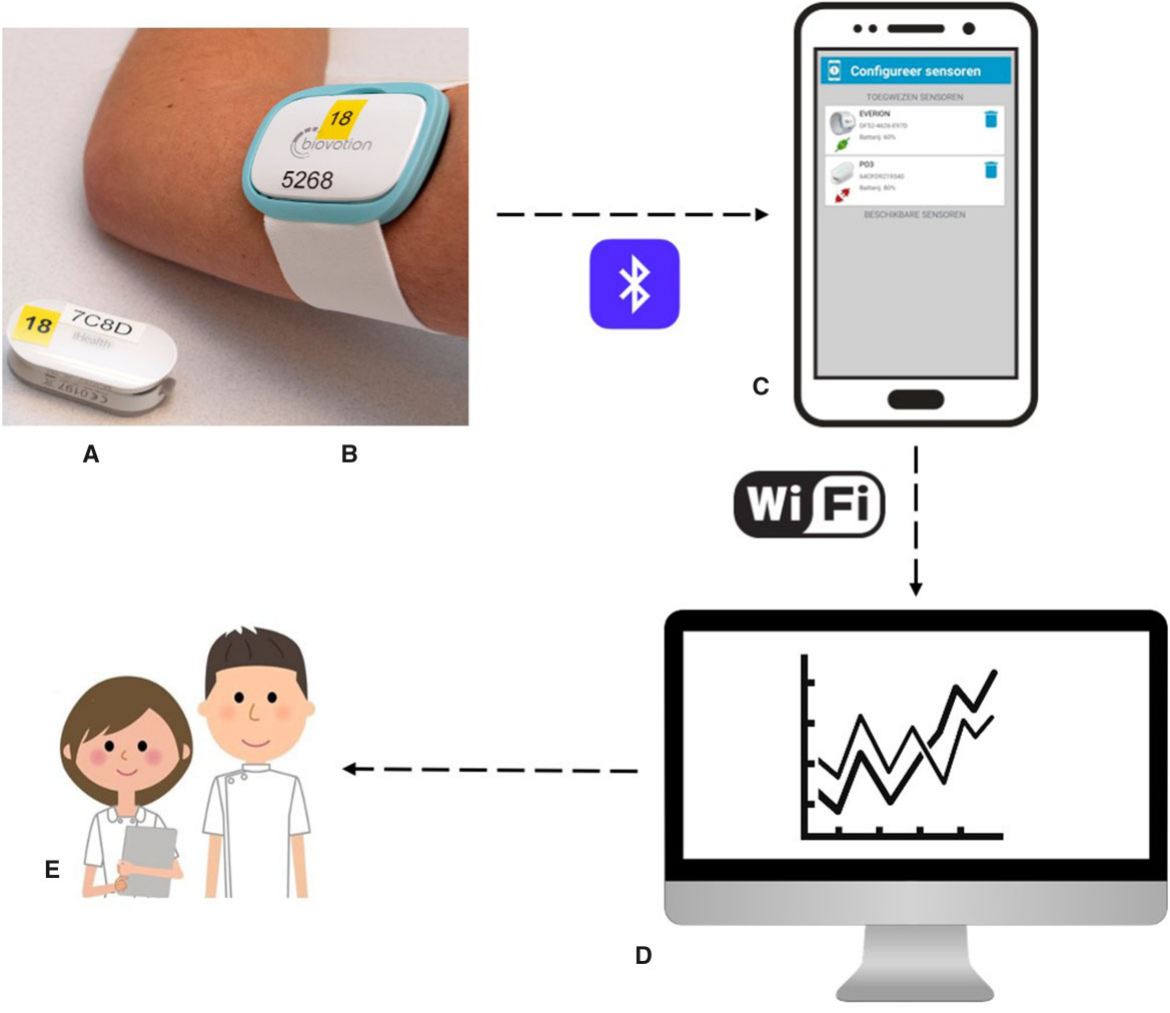
Overview of the data transmission. (A) Biovotion Everion, (B) iHealth Pulse Oximeter, (C) mobile device, (D) multisensor platform Digistat, and (E) nursing staff on the COVID-19 ward.

All patients who had been tested positive for SARS-CoV-2 and admitted to our hospital were considered at risk for rapid clinical deterioration and were connected to 2 wireless sensors (Figure 1). Vital parameters obtained by these wireless systems were 24/7 monitored by a trained operator at distance. Also, heart rate (HR), respiratory rate, temperature, blood pressure, level of consciousness, urine production, and oxygen saturation were measured at bedside at least 3 times a day. The collected data were retrospectively analyzed after formal approval for this study had been given by the local medical ethical committee.

### Study population

All patients admitted to the COVID-19 ward of our hospital between April 1 and May 15, 2020 were considered for inclusion. Impaired cognition and/or psychiatric illness were defined as exclusion criteria. Monitoring was discontinued if patients had to be transferred to the intensive care for further treatment or in case palliative care was started. Patients were excluded from further analyses if subsequent PCR did not test positive for SARS-CoV-2.

### Wireless devices and data collection

HR and respiratory rate were recorded every second using the Everion sensor (Biovotion AG, Zürich, Switzerland). Saturation was recorded every fifteen minutes using the iHealth Labs Pulse Oximeter (iHealth Labs Inc., Mountain View, CA, USA). Patients were allowed to ambulate while wearing the devices. However, most patients remained in bed due to physical impairment.

Data from these devices were transmitted to a mobile device via Bluetooth. Wi-Fi facilitated data transmission from the mobile device to a secured multisensor platform (Digistat) developed by Ascom (Figure 1). A link between the Digistat platform and the electronic medical record (EMR) and a mobile notification device allowed real-time insight in the patients vital signs. All data registered by the Everion and the Pulse Oximeter were eventually retrieved from the Ascom’s Digistat platform (Ascom Holding AG, Baar, Switzerland) for further analysis. Other patient data were manually collected from the hospital’s EMR.

#### Study procedure and automated early warning score

An aEWS was calculated every 5 minutes using heart- and respiratory rate (Table 1). aEWS values of less than 3 were considered to be normal. To correct for outliers, the aEWS was calculated using the median values of both heart and respiratory rate within a 5-minute window. Oxygen saturation values were not included in the aEWS because they were only measured every 15 minutes. The aEWS is based on the EWS used in our hospital’s RRT activation protocol. Trained operators were situated in a control room, where they remotely monitored the vital signs of all patients and noticed elevated aEWS alarms. The protocol for alerting nursing staff was based on the existing COVID-19 literature at that time and the expertise of our pulmonologists. Nursing staff were alerted if a patient exhibited 2 or more elevated aEWS’s in a time span of half an hour or if oxygen saturation dropped below 90% or dropped more than 4%. Nursing staff were then instructed to check on the patient and if deemed necessary perform extra measurements or alert the attending physician. All vital signs and aEWS scores were also accessible for the nursing staff itself via the hospital’s EMR.

**Table 1.** Automated early warning score (aEWS)

Score	3	2	1	0	1	2	3
Respiration rate (p/min)		<9		9–14	15–20	21–30	>30
Heart rate (p/min)		<40	40–50	51–100	101–110	111–130	>130

The aEWS is calculated based on respiration rate and heart rate. Both parameters have an associated score ranging from 0 to 3. The total score is the sum of both components.

### Data analysis

Patients were divided into 2 groups depending on whether adverse events had occurred. Adverse events were defined as transfer to intensive care unit (ICU) or death. Normally distributed continuous variables are presented as mean ± standard deviation, whereas non-normally distributed continuous or ordinal variables are expressed as median and interquartile ranges (IQR). Differences were tested using a Student’s *t* test and Mann–Whitney *U* test for the respective variables. For categorical and dichotomous variables, Fisher’s exact test was used to evaluate differences between groups. Density and Q–Q plots were used to assess normality. Analyses were adjusted for multiple comparisons using the Benjamini–Hochberg procedure and the resulting *q* values are reported along with the original *P* values. A *q* value <0.05 was considered significant.

To provide a confidence interval to our results, we report a 95% confidence interval of the Mann–Whitney parameter as described in “Confidence intervals of the Mann–Whitney parameter that are compatible with the Wilcoxon–Mann–Whitney test” [9].

The Mann–Whitney parameter is consistent with the Mann–Whitney *U* test. This parameter can be understood as the probability that a random individual from the adverse event group has a greater value (eg, mean automated EWS) than a random individual from the other group.

We compared multiple derived parameters between groups. For every patient mean aEWS, median automatic EWS, median SPO_2_, median HR, median respiration rate (RR), and the average number of automatic EWS ≥3 per day were calculated.

## RESULTS

Between the April 1 and May 4, 2020, 34 patients were enrolled in this study, of whom 6 were eventually excluded due to a subsequent negative PCR test. Serious adverse events were seen in half of all cases (*n* = 14); 5 patients were transferred to ICU and 10 patients died. Because of limited ICU capacity 1 patient was transported to a nearby hospital and subsequent data were lost. Patient characteristics are shown in Table 2. The median age was 69.5 years in the adverse events group and 66.0 in the control group.

**Table 2.** Patient characteristics

Parameter	Patients without adverse events (*n *=* *14)	Patients with adverse events(*n *=* *14)	*P* value
Age, median (IQR) (years)	66 (57.25–75.75)	69.5 (64.25–81.25)	0.290
Male gender, *n* (%)	6 (42.9)	6 (42.9)	1.000
BMI, median (IQR)	25.68 (24.82–26.80)	27.15 (24.54–32.62)	0.251
Comorbidities			
Hypertension, *n* (%)	5 (35.7)	8 (57.1)	0.449
Diabetes, *n* (%)	1 (7.1)	6 (42.9)	0.081
Respiratory disease, *n* (%)	3 (21.4)	5 (35.7)	0.676
Cardiovascular disease, *n* (%)	3 (21.4)	7 (50.0)	0.237
COVID-19 medication			0.408
Ceftriaxone, *n* (%)	4 (28.6)	7 (50)	
Hydroxychloroquine + cefuroxime, *n* (%)	7 (50.0)	6 (42.9)	
Chloroquine + cefuroxime, *n* (%)	1 (7.1)	1 (7.1)	
No medication, *n* (%)	2 (14.3)	0 (0.0)	
CO-RADS classification, median (IQR)	5 (4.00–5.00)	5 (4.75–5.00)	0.806
Length of stay, median (IQR)	11.50 (9.25–13.75)	8.00 (5.00–11.00)	0.046
Time on wireless devices, median (IQR)	7.25 (5.64–10.03)	2.10 (1.06–2.65)	<0.001

Data are presented as absolute number *n* (%) or median (interquartile range).

### Main outcomes

Main outcomes are presented in Table 3. Patients who eventually developed adverse events had significantly higher mean automated EWS values, median automated EWS values, median HRs, and median RRs prior to the occurrence of the event. Patients with adverse events had also significantly lower median SPO2 values. Consequently, patients in the adverse events group had significantly more automated EWS alarms per day.

**Table 3.** Outcome statistical analyses per parameter

Parameter	Patients without adverse events, median (IQR)	Patients with adverse events, median (IQR)	*P* value	*Q* value	Mann–Whitneyestimate	95% confidenceinterval
Mean aEWS	1.38 (1.28–1.77)	2.03 (1.76–2.32)	0.0022	0.0100	0.84	0.63–0.94
Median aEWS	1.5 (1–2)	2 (2–2)	0.0201	0.0321	0.71	0.53–0.84
Median HR	75.5 (68.25–77.75)	80.5 (78.25–92.75)	0.0138	0.0276	0.78	0.56–0.9
Median RR	20 (19.25–23)	25 (23–26)	0.0025	0.0100	0.83	0.62–0.93
Median SpO_2_	93.5 (92.25–94)	90 (87.25–91.88)	0.0069	0.0184	0.20	0.09–0.41
Average number aEWS ≥3 per day	6.9 (1.61–11.2)	21.9 (10.85–73.5)	0.0149	0.0238	0.77	0.55–0.9

Data are presented as median (IQR).

Mann–Whitney parameter estimates differed significantly from 0.5 for mean automated EWS, median automated EWS, median HR, median RR, median SPO_2_, and average number of automated EWS ≥3 per day.

## DISCUSSION

Our findings highlight the possibility of early deterioration prediction using continuous monitoring in COVID-19 patients. The limited amount of available intensive care beds and the unpredictable course of this new disease, highlights the relevance for deterioration predictors in COVID-19 care. We believe our main finding is the significance of the difference in vital signs measurements between patient with and without severe adverse events. The numerical difference was less relevant due to variability between sensors. COVID-19 patients transferred to intensive care or those who died showed changes in HR, respiratory rate, and oxygen saturation prior to the occurrence of adverse events. Although differences between groups were found, the number of aEWS is quite substantial for both patients with or without severe adverse events. This may be the effect of the severity of COVID-19 causing more fluctuation of vital signs resulting in more aEWS alarms compared with other conditions. Evaluation of the sensitivity of the aEWS alerting therefore requires further evaluation in another patient population.

### Strength and limitations

To our best of knowledge, no data have been published about continuous monitoring of COVID-19 patients. Previous studies in other patient populations frequently tested only 1 device and did not have an automated alarm system incorporated [3–5]. We believe future medical care will be increasingly dependent on automatic measurements and it will be vital to have adequate systems in place to handle the data efficiently. Alarm fatigue is a well-documented symptom and it is important for patient safety to keep the amount of alarms at a minimum [10]. Ruskin and Hueske-Kraus [11] found that alarm fatigue can be countered with appropriate thresholds and directing of alarms. Therefore, to prevent additional strain of working with new technology on nurses, we have incorporated a control room where the alarms were routed and vital signs were monitored. These trained operators had more time to review data, were better able to see trends, and decreased the amount of false-positive alarms. We found a control room to be a feasible and scalable solution for future endeavors, while better algorithms might decrease the added value in the future.

The implementation of this new system presented some challenges. Although possible, most pulse oximeters are not intended to be used for continuous monitoring. Wireless pulse oximeter sensors showed to be prone to failure after long periods of use. With technical and practical solutions it is possible to minimize the downtime of these sensors. Other causes of missing data from both the saturation finger sensor and the arm sensor were attributed to low batteries and connectivity (WiFi and Bluetooth) problems.

Due to the non-normality of our data, we used the Mann–Whitney *U* test to assess difference in mean ranks between groups. We used the techniques described in “Confidence intervals of the Mann–Whitney parameter that are compatible with the Wilcoxon–Mann–Whitney test” [9] to provide confidence intervals to our results. The paper introduces the notion of the Mann–Whitney parameter. To illustrate how the Mann–Whitney parameter should be interpreted, we shall take a closer look at the parameter estimate for mean automated EWS. The Mann–Whitney parameter estimate is 0.84 with a corresponding 95% confidence interval ranging from 0.63 to 0.94. This means that based on our data, 95 of 100 times the true probability that a random patient from the adverse event group has a greater mean automated EWS than another random patient from the nonadverse event group, is contained within the range 0.63–0.94.

The system we used in this study is a first iteration, it will be improved and evaluated for neurological and vascular patients in our hospital in a prospective study. Future research should focus on additional vital parameters, different sensors and advanced pattern recognitions. While trained operators were able to spot trends, we were unable to quantify these changes in this study. Furthermore, once a robust system is available, the focus of research should shift to improving clinical outcomes with the implementation of that system. In COVID-19 care, this system has proven to be a valuable risk screening tool in addition to standard care.
